# A Common Role for Various Human Truncated Adenomatous Polyposis Coli Isoforms in the Control of Beta-Catenin Activity and Cell Proliferation

**DOI:** 10.1371/journal.pone.0034479

**Published:** 2012-04-03

**Authors:** Shree Harsha Vijaya Chandra, Ingrid Wacker, Uwe Kurt Appelt, Jürgen Behrens, Jean Schneikert

**Affiliations:** Nikolaus-Fiebiger-Center for Molecular Medicine, University of Erlangen-Nürnberg, Glückstrasse, Erlangen, Germany; Cardiovascular Research Institute Maastricht - Maastricht University, The Netherlands

## Abstract

The tumour suppressor gene adenomatous polyposis coli (APC) is mutated in most colorectal cancer cases, leading to the synthesis of truncated APC products and the stabilization of β-catenin. Truncated APC is almost always retained in tumour cells, suggesting that it serves an essential function. Here, RNA interference has been used to down-regulate truncated APC in several colorectal cancer cell lines expressing truncated APCs of different lengths, thereby performing an analysis covering most of the mutation cluster region (MCR). The consequences on proliferation in vitro, tumour formation in vivo and the level and transcriptional activity of β-catenin have been investigated. Down-regulation of truncated APC results in an inhibition of tumour cell population expansion in vitro in 6 cell lines out of 6 and inhibition of tumour outgrowth in vivo as analysed in one of these cell lines, HT29. This provides a general rule explaining the retention of truncated APC in colorectal tumours and defines it as a suitable target for therapeutic intervention. Actually, we also show that it is possible to design a shRNA that targets a specific truncated isoform of APC without altering the expression of wild-type APC. Down-regulation of truncated APC is accompanied by an up-regulation of the transcriptional activity of β-catenin in 5 out of 6 cell lines. Surprisingly, the increased signalling is associated in most cases (4 out of 5) with an up-regulation of β-catenin levels, indicating that truncated APC can still modulate wnt signalling through controlling the level of β-catenin. This control can happen even when truncated APC lacks the β-catenin inhibiting domain (CiD) involved in targeting β-catenin for proteasomal degradation. Thus, truncated APC is an essential component of colorectal cancer cells, required for cell proliferation, possibly by adjusting β-catenin signalling to the “just right” level.

## Introduction

The stimulation of the canonical wnt pathway by wnt growth factors leads to the activation of a genetic program controlling the coordinated expansion, fate and sorting of the epithelial cell population of the colon. The wnt signalling cascade induces the stabilisation of β-catenin which contributes to the transcription of specific target genes [1], [2], [3]. The levels of cytoplasmic β-catenin are normally controlled by a multiprotein destruction complex which is assembled over the tumour suppressor APC [4]. The destruction complex promotes phosphorylation of β-catenin, which is subsequently degraded in the proteasome [2]. In the presence of Wnt, the destruction complex is inactivated, resulting in the stabilization of β-catenin.

**Figure 1:**
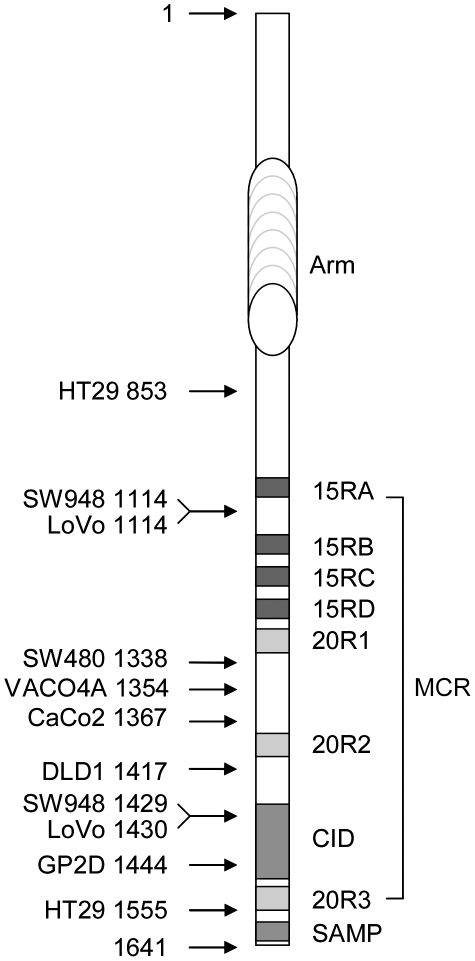
Truncated APC products from SW480, DLD1, HT29, GP2D, CaCo2, LoVo, SW948 and VACO4A cells. Functional domains of APC are indicated and include the Armadillo repeat (Arm) domain, the 15 (15RA–15RD) and 20 (20R1–20R3) amino acid repeats that are β-catenin binding sites, the β-catenin inhibitory domain (CID) which is necessary to target β-catenin for degradation and the first axin/conductin binding site (SAMP). The mutation cluster region (MCR) extends from the end of the 15RA to the middle of the 20R3 and corresponds to the region where most mutations associated with colorectal cancer have been found. The indicated amino acid positions refer to the length of the various truncated APCs expressed in the different colorectal cancer cell lines. SW480, DLD1, GP2D, CaCo2 and VACO4A cells have a truncating mutation at one allele and underwent loss of heterozygocity at the second allele, whereas SW948, HT29 and LoVo cells carry each two different truncating mutations.

In colorectal cancer, epithelial cells proliferate inappropriately because they acquired mutations in components of the wnt pathway, therefore mimicking the effect of a permanent Wnt stimulation [2]. APC mutations occur in a high proportion of sporadic colorectal carcinomas (up to 80%) and were first identified in the germline of FAP (familial adenomatous polyposis) patients [5], [6]. Several studies have shown that APC inactivation in vivo in mice is sufficient to initiate adenoma development [7], [8], [9], [10], [11]. Mutations of the APC gene affect both alleles and occur mostly in a mutation cluster region (MCR) which is located approximately in the middle of the open reading frame (fig. 1). These mutations generate stop codons or frameshifts leading to the deletion of the C-terminal half of the APC protein. It is commonly accepted that the major consequence of APC mutations with respect to wnt signalling is the failure of assembling a functional β-catenin destruction complex which ultimately results in the constitutive stabilization of β-catenin.

In addition to this negative selection, colorectal cancer cells almost invariably retain at least one truncated APC product whose length is defined by the position of the MCR and, occasionally, a second but shorter product (fig. 1) [12]. The reason for this retention is not clear, but the strong selection for the presence of truncated APC indicates that it must fulfil an important function. Actually, the “just right signalling” hypothesis [13] for which experimental support has been recently provided [9], [14] states that APC truncating mutations are selected to avoid too much β-catenin signalling that would be detrimental to tumour development. Our previous data have shown that down-regulation of APC in SW480 cells results in a stimulation of the β-catenin transcriptional activity accompanied by a reduction of cell proliferation [15]. This indicates that truncated APC in SW480 cells serves an essential function. Human tumours, however, express truncated APCs of different lengths, having retained different functional domains, i.e. the β-catenin inhibitory domain (CiD) and part of the third 20 amino acid repeat, that provide variable efficiencies in targeting β-catenin for degradation [16], [17]. It is not clear whether the observations made in SW480 cells represent a fortuitous event or can be generalized throughout the MCR. It is also not known whether truncated APC displaying the CiD can still control the level of β-catenin under endogenous settings. In the present work, we used RNA interference [18] to down-regulate the level of APC in several colon cancer cell lines expressing truncated APCs of different lengths covering the MCR. The consequences on the level and transcriptional activity of β-catenin, proliferation in vitro and tumour formation in vivo were investigated.

## Results

### Truncated APC controls proliferation of colorectal cancer cell lines and β-catenin activity

The first step of our analysis consisted in asking whether APC would constitute an essential component required for cell proliferation in vitro. For that purpose, we took advantage of the availability of a siRNA directed against APC for which we had already shown the efficiency [15]. This siRNA sequence was expressed as a small hairpin structure (N-APC shRNA) from a lentiviral vector [19] co-expressing also GFP (shN-APC, see Methods). As a negative control, we used the empty viral vector (shVEC). To investigate the possibility of achieving an allele-specific down-regulation of APC, we also designed a lentivirus encoding a shRNA specific for the APC isoform expressed by VACO4A cells that does not recognize wild-type APC or truncated APC isoforms from the other colorectal cancer cell lines (shVACO4A, see methods). The resulting constructs were packaged into a lentiviral capsid to transduce colorectal cancer cell lines. Transduced cells having stably integrated the lentiviral provirus into their genome and therefore expressing GFP were isolated by fluorescence activated cell sorting (FACS), replated and analysed for APC expression and a possible effect on cell population expansion.

**Figure 2:**
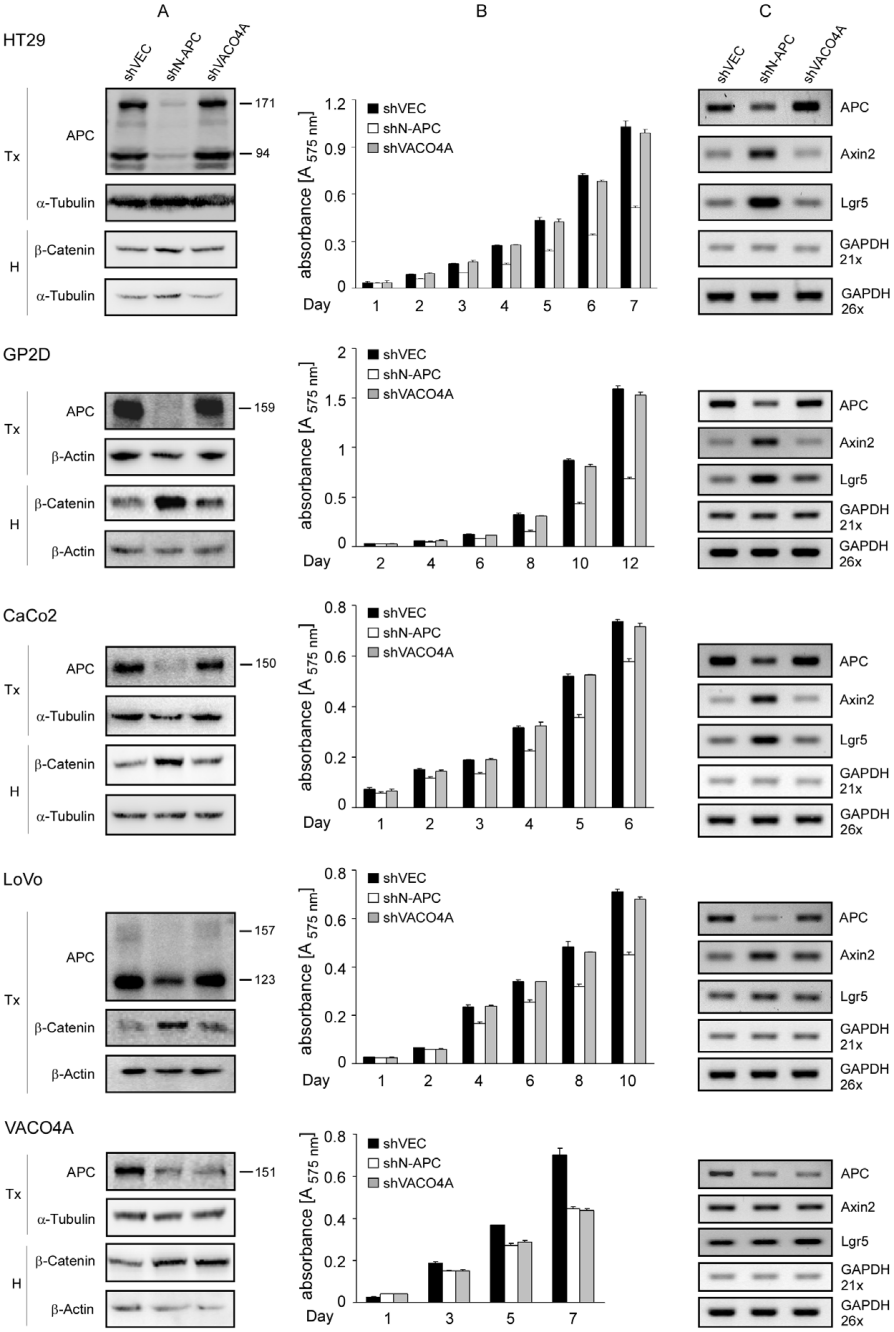
Stable knockdown of APC reduces colorectal cancer cell proliferation in vitro and increases wnt signalling. HT29, GP2D, CaCo2, LoVo and VACO4A cells were transduced with lentiviruses for the expression of either shVEC, shN-APC or shVACO4A and the transduced cells were sorted by FACS based on GFP co-expressed by the lentivirus. Data are representative of at least two independent experiments. (**A**) Efficiency of APC knockdown and β-catenin level as determined by western blotting in Triton-X100 (Tx) or hypotonic (H) cell lysates. α-tubulin and β-actin were used as controls for equal sample loading. Molecular weights of APC isoforms (kD) are indicated. (**B**) Effect of APC knockdown on cellular proliferation, analyzed using the colorimetric MTT assay over the indicated time course. Data are the mean of triplicates+/−standard deviation. (**C**) Semi-quantitative RT-PCR of APC, axin2, Lgr5 and GAPDH (21 and 26 cycles).

**Figure 3:**
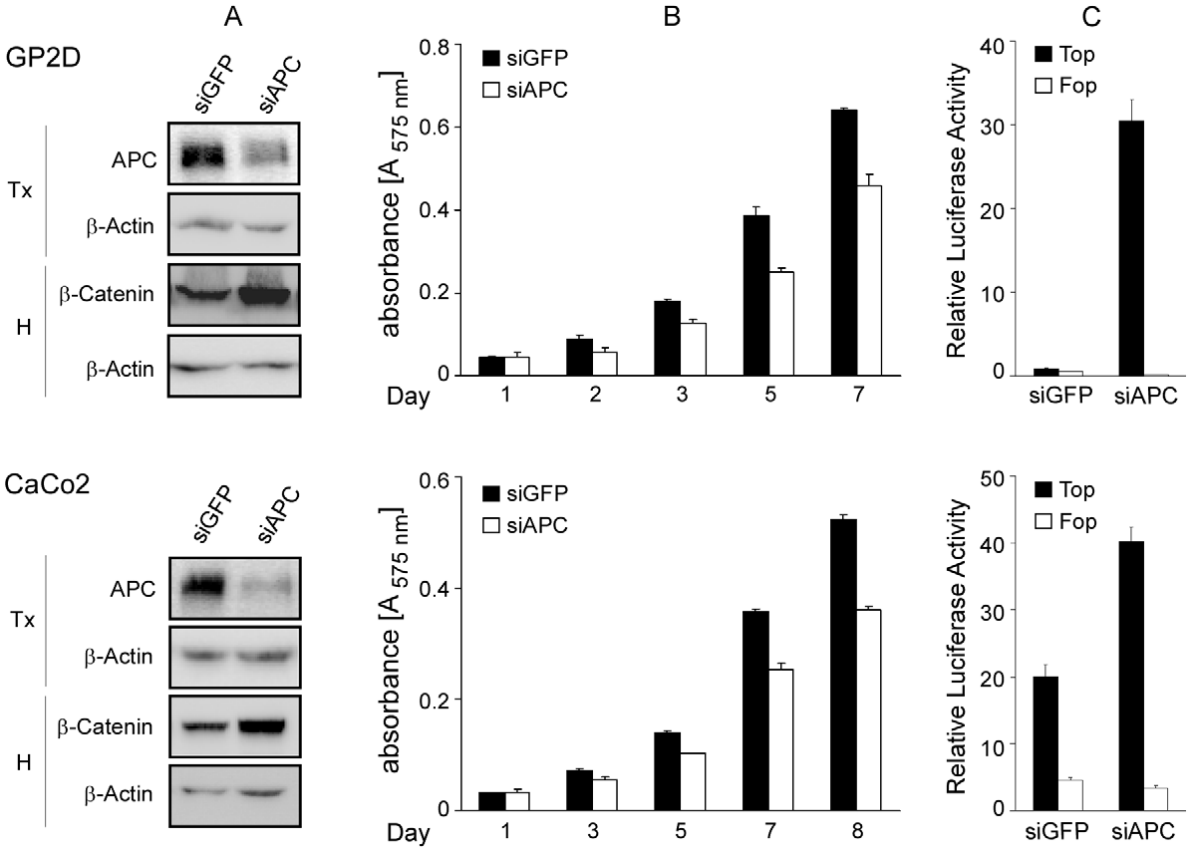
Transient knockdown of APC reduces colorectal cancer cell proliferation in vitro and increases wnt signalling. GP2D and CaCo2 cells were transiently transfected with either siGFP or siAPC and further processed two days after transfection. Data are representative of at least two independent experiments. (**A**) Efficiency of APC knockdown and β-catenin level. (**B**) Effect of APC knockdown on cellular proliferation, as described in legend to figure 2B. (**C**) β-catenin-dependent reporter assay. Cells were transiently transfected with either the TOPglow or control FOPglow reporters, together with a β-galactosidase expression vector to correct for variations in the transfection efficiency. Luciferase activity was measured 48 h post-transfection and normalized to β-galactosidase values. Data are represented as the mean value of triplicates+/−standard deviation. To visualize FOP activity, values have been multiplied by 4 and 100 for GP2D and CaCo2 cells, respectively.

**Figure 4:**
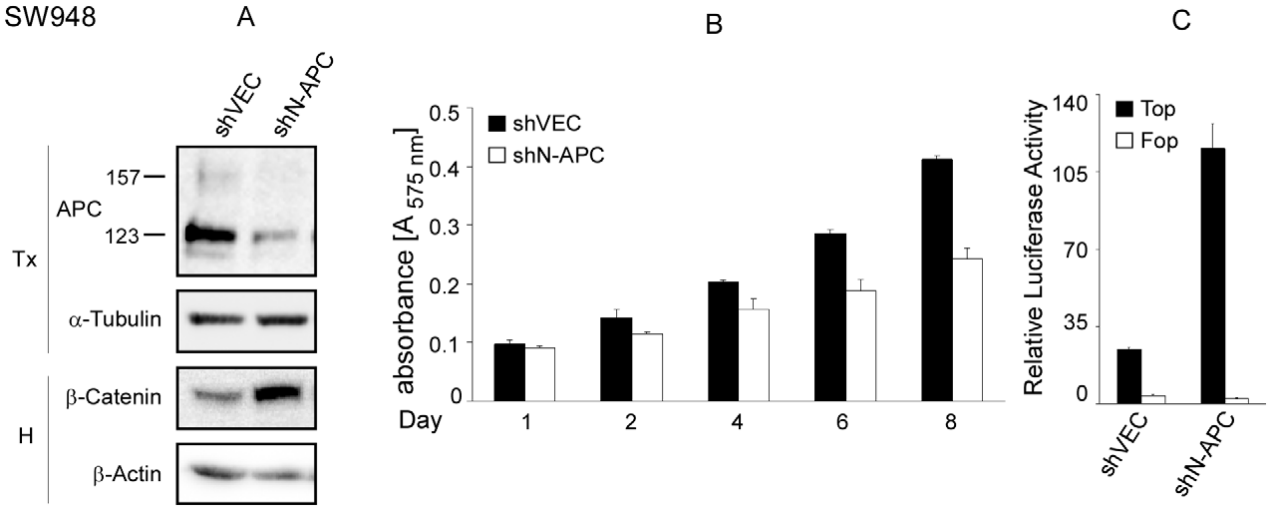
Knockdown of truncated APC reduces proliferation of SW948 cells in vitro and increases wnt signalling. SW948 cells were transduced with lentiviruses for the expression of either shVEC or shN-APC and the transduced cells were sorted by FACS based on GFP co-expressed by the lentivirus. Data are representative of at least two independent experiments. (**A**) Efficiency of APC knockdown and β-catenin level. (**B**) Effect of APC knockdown on cellular proliferation, as described in legend to figure 2B. (**C**) β-catenin-dependent reporter assay, as described in legend to figure 3C. To visualize FOP activity, values have been multiplied by 10.

The N-APC shRNA inhibited the expression of APC protein (fig. 2A) and mRNA (fig. 2C) in HT29, GP2D, CaCo2 and LoVo cells. In contrast, the empty viral vector shVEC or the one encoding the VACO4A shRNA, which serve as a control, did not affect the levels of APC in these cells. The consequences of APC down-regulation were investigated in a cell proliferation assay measuring the relative numbers of live cells. The N-APC shRNA, but not the VACO4A shRNA, inhibited the expansion of the populations of HT29, GP2D, CaCo2 and LoVo cells (fig. 2B). APC down-regulation was associated with an increase of β-catenin level in GP2D, CaCo2 and LoVo cells, but not in HT29 cells (fig. 2A). Fractionation of GP2D and LoVo cells revealed an increase of nuclear β-catenin upon APC down-regulation (fig. S1). This did not occur in HT29 cells (fig. S1). The transcriptional activity of β-catenin, as measured by semi-quantitative RT-PCR of the β-catenin target genes axin2 [20], [21] and Lgr5 [22] increased in the cell lines transduced with the shN-APC, but not the shVACO4A (fig. 2C). These effects were not due to an off-target effect of the shRNA, because transient transfection of an unrelated APC siRNA [15] in GP2D and CaCo2 cells also led to reduced cell proliferation and β-catenin-dependent transcription as measured by a luciferase-based reporter assay (see Methods) (fig. 3). Finally, we could also show that the N-APC shRNA inhibited efficiently the expression of both truncated APCs present in SW948 cells (fig. 4A), led to an up-regulation of the amount of β-catenin (fig. 4A), attenuated cell proliferation (fig. 4B) and stimulated the transcriptional activity of β-catenin (fig. 4C). Together, our results indicate that truncated APC is required for the expansion of colorectal cancer cells in vitro, in line with a previous observation made in SW480 cells [15], and that truncated APC suppresses not only β-catenin signalling but surprisingly also its protein level.

The VACO4A specific shRNA inhibited APC expression in VACO4A cells (fig. 2A), which was accompanied by a slight increase of the β-catenin level (fig. 2A) and a reduced expansion of the VACO4A cell population (fig. 2B). The observed effects were likely due to APC down-regulation, because they also occurred with the unrelated N-APC shRNA. We did not observe any significant modification of axin2 and Lgr5 transcripts levels (fig. 2C). The VACO4A shRNA did not affect the APC level in HT29, GP2D, CaCo2 and LoVo cells. These cells express full length APC mRNAs that are wild-type at the sequence mutated in VACO4A cells and can thus be taken as surrogates of wild-type APC. Also, the VACO4A shRNA did not affect the APC level in HCT116 cells that express the wild-type APC mRNA (fig. S2). Thus, our data show that it is possible to design an allele-specific siRNA that distinguishes a deletion from the wild-type allele. We conclude that the expansion of colorectal cancer cell lines can be inhibited by RNA interference against APC in an allele-specific manner.

### Truncated APC controls tumour development

**Figure 5:**
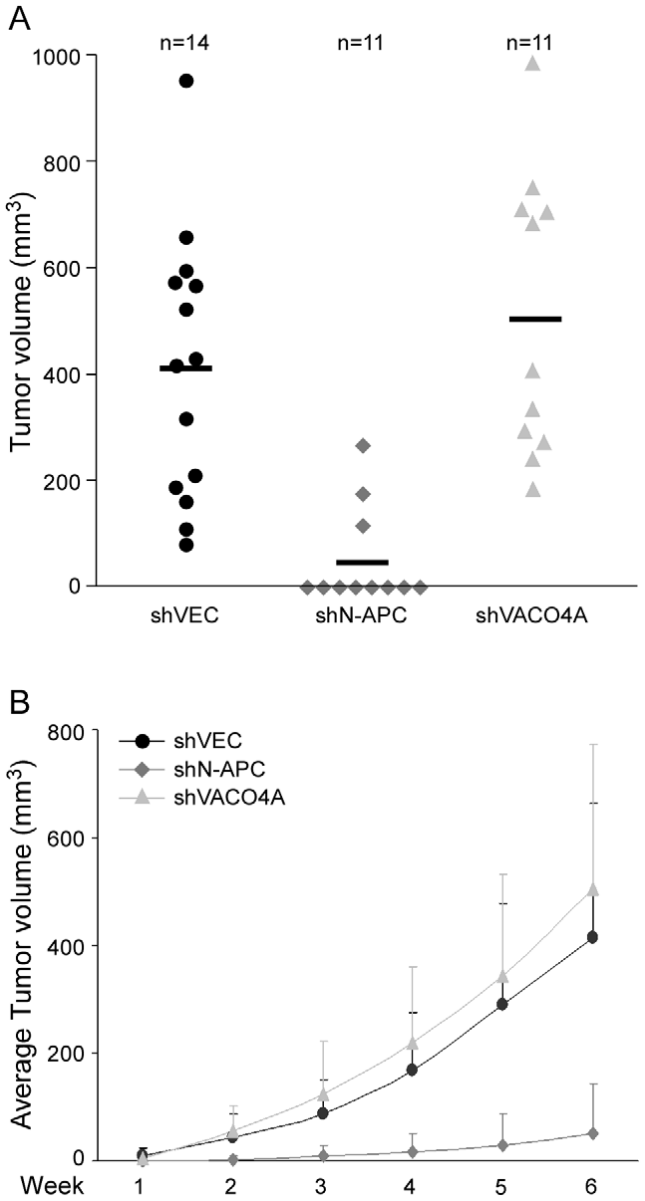
Knockdown of truncated APC inhibits tumour formation by HT29 cells in nude mice. Lentivirus transduced HT29 cells expressing either shVEC, shN-APC or shVACO4A were injected subcutaneously into both flanks of nude mice (n, number of injections). Tumours were measured once a week over a period of 6 weeks post injection. (**A**) Tumour volumes upon termination shown as a dot plot. Bar, mean tumour size. (**B**) Increase of the mean tumour volume over time, +/−standard deviation.

In the next step, we asked whether a reduction of the APC level in colorectal carcinoma cells would also inhibit the formation of tumours in nude mice. Therefore, HT29 cells transduced with the control virus shVEC and those encoding either the N-APC shRNA or the VACO4 shRNA were sorted and injected subcutaneously into nude mice. The experiment showed that HT29 cells transduced with the control virus formed tumours of variable sizes within a period of about six weeks, after which mice were sacrificed (fig. 5A). HT29 cells expressing the N-APC shRNA developed only few tumours, which had a relatively modest volume (fig. 5A) and grew slower than control tumours (fig. 5B). In contrast, tumours produced by HT29 cells expressing the VACO4A specific shRNA grew at frequencies and sizes comparable to control tumours. We conclude that truncated APC in HT29 cells is required for tumourigenesis.

## Discussion

Knockdown of truncated APC reduced the in vitro proliferation of 6 out of 6 human colorectal cancer cell lines (**table 1**) and inhibited tumour development by HT29 cells upon transplantation in nude mice. A more detailed analysis revealed that down-regulation of truncated APC in all 6 cell lines did not correlate with increased apoptosis, by measuring the extent of proteolytical cleavage of the executioner of apoptosis caspase 3 and its substrate poly (ADP-ribose) polymerase [23] (**data not shown**). Our results extend previous observations showing that truncated APC from SW480 cells [15] and full length APC from HCT116 [24] cells are required for cell proliferation in vitro. The “scan” analysis we performed along the MCR by investigating the role of APCs of different lengths indicates that truncated APC is still under selective pressure in the cell lines and provides a general explanation as to why colorectal cancer cells invariably retain expression of truncated APC despite their structural variability.

**Table 1:** Consequences of APC down-regulation.

Cell line (amino acid position of APC truncation)	β-catenin level	β-catenin activity	proliferation
SW480 (1338+LOH)	not changed^1^	up^1^^,^ 2	down^4^
VACO4A (1354+LOH)^5^	up	not changed	down
CaCo2 (1367+LOH)^5^	Up	up^2^^,^ 3	down
DLD1 (1417+LOH)	not changed^1^	up^1^^,^ 2	not done
LoVo (1429+1114)^5^	Up	up^3^	down
SW948 (1430+1114)^5^	Up	up^2^	down
GP2D (1450+LOH)^5^	Up	up^2^^,^ 3	down
HT29 (1555+853)^5^	not changed	up^3^	down

LOH, loss of heterozygocity.

1 , reference [25].

2 , as measured by using the TOP/FOP assay.

3 , as measured by RT-PCR of the axin2 and Lgr5 target genes.

4 , reference [15].

5 , this study.

Why is truncated APC required for tumour development in humans? The combined data from current and previous studies (**summarized in** **table 1**) indicate that APC down-regulation is associated with a stimulation of the β-catenin transcriptional activity in SW480 [25], DLD1 [25], CaCo2, LoVo, SW948, GP2D and HT29 cells. VACO4A cells represent an exception in the sense that the levels of axin2 and Lgr5 mRNAs were not affected by APC down-regulation in these cells. However, we cannot exclude that the transcription of these β-catenin target genes is already at a maximum in VACO4A cells, but that other genes are still sensitive to a modification of the β-catenin level. Thus, it is tempting to hypothesize that colorectal tumours always retain a truncated APC product to still keep a control over the transcriptional activity of β-catenin and avoid it to reach a level too high, detrimental for tumour growth, in accordance with the “just right signalling” model [13]. Alternatively, truncated APC might be required for tumour development independently of its control over the transcriptional activity of β-catenin, as previously discussed [15].

How is truncated APC still able to regulate the transcriptional activity of β-catenin? Our data indicate that truncated APC can influence the transcriptional activity of β-catenin by at least two different mechanisms. In HT29 cells, we observed a stimulation of the transcriptional activity of β-catenin upon APC down-regulation without any obvious increase of the β-catenin level. Truncated APC from HT29 cells has retained not only the CiD but also the third 20 amino acid repeat, both together conferring a strong β-catenin down-regulating activity to APC [16], [17], [26]. It is therefore not surprising that lowering the APC level in HT29 cells does not lead to an increase of the β-catenin concentration. However, the reduction of the APC level in HT29 cells may result in a higher concentration of “free” β-catenin available for transcription despite the fact that the overall level of β-catenin remains constant. This possible titration of β-catenin exerted by truncated APC may likely involve the 15 and/or 20 amino acid repeats (fig. 1) [25]. Note however that in the HT29 cell line, the amount of nuclear β-catenin does not increase upon APC down-regulation (fig. S1), which is speaking against a mechanism of cytoplasmic retention of β-catenin by truncated APC [27]. Alternatively, the down-regulation of APC may promote the activity of other signalling pathways that ultimately lead to β-catenin-dependent transcription. For example, it has been shown that APC down-regulation in CBS and Moser colon cancer cells converts the negative effect of TGFβ on wnt signalling into a positive one, without altering the amount of nuclear β-catenin [28]. Similarly, it has been reported that APC knock-down stimulates PAK1-mediated β-catenin phosphorylation on Ser675, leading to a weaker interaction of β-catenin with HDAC1 but to a stronger interaction with CBP, thereby resulting in enhanced β-catenin transcriptional activity [29], [30]. APCs from GP2D and the long APC isoforms from LoVo and SW948 cells have retained the CiD that provides a β-catenin down-regulating activity [16], [17] and therefore a likely explanation for the up-regulation of both the level and the transcriptional activity of β-catenin after APC knock-down. APCs from DLD1, CaCo2, VACO4A and SW480 cells lack the CiD and representative constructs with a similar length cannot target β-catenin for destruction upon transient ectopic expression [16]. Our data indicate however that truncated APCs from CaCo2 and VACO4A still have retained some residual β-catenin down-regulating activity. The associated molecular mechanism is not clear and might be related to the stimulation of β-catenin phosphorylation, the stabilization of phosphorylated β-catenin or the transfer to the ubiquitination machinery [31]. Truncated APCs from DLD1 and SW480, described in previous studies [15], [25], do not apparently control the β-catenin level. The discrepancy relative to VACO4A and CaCo2 cells may first depend on cell-specific mutational events (other than APC) in these cells that are from different origins. Second, one cannot exclude that it depends on the specific nature and/or location of the APC mutations along the sequence. Third, different β-catenin and APC intracellular concentrations, as well as different β-catenin to APC ratios between the cell lines, together with the extent of APC down-regulation may also explain why down-regulation of truncated APCs in SW480 or DLD1 cells does not affect the level of β-catenin. For instance, SW480 cells contain higher amounts of signalling (not membrane-bound) β-catenin relative to the other cell lines (fig. S3) and it is possible that a further accumulation cannot be achieved or tolerated. Inversely, DLD1 cells express high amounts of truncated APC (fig. S3) and it is possible that its down-regulation was not efficient enough to reveal an effect on the β-catenin level. Meanwhile, truncated APCs from SW480 and DLD1 cells still modulate the transcriptional activity of β-catenin, possibly by one of the mechanisms described above for HT29 cells.

Germ-line deletions of the whole APC gene in FAP patients have been described earlier [32], [33], but the nature of the somatic hit that follows the germ-line deletion is not known. These patients had developed an intermediary type of FAP characterized by the occurrence of approximately 1000 polyps. A genotype-phenotype correlation analysis [34] suggests the presence of a mutation in the MCR of the second APC allele in the polyps. First, the phenotype is very similar to the one of patients harbouring a germ-line mutation before the MCR and in whom the development of polyps is dictated by the acquisition of an optimal truncating somatic mutation in the MCR of the second allele. Second, the phenotype associated with a germ-line APC whole gene deletion is contrasting with the profuse phenotype characterized by a higher number of polyps (∼5000) and LOH consecutive to a germ-line mutation in the MCR. Thus, if APC was not necessary for tumour development in patients with a germ-line deletion of the APC gene, one would expect loss of heterozygocity (LOH) as the mechanism of inactivation of the second APC allele, because it is spontaneously more frequent than a mutation [35]. As a consequence, the patients with a germ-line APC deletion would have presented with the profuse phenotype, which was not the case. It is therefore likely that a germ-line APC whole gene deletion is followed by a mutation in the MCR of the second allele, a hypothesis which is also supported by our results indicating a requirement of truncated APC in tumour cells.

Our data contradict those obtained in the mouse showing that polyposis can initiate after a complete loss of both APC alleles [36]. Several other mice models of intestinal cancers have also been created, by introducing constitutive truncating mutations of APC in the germ-line [8], [9], [10], [11] (for a review, see [37]). Though developing intestinal adenomas, these models present however two striking differences to the human disease, that are independent of the location of the germ-line mutation in the mouse APC sequence. In the mouse, adenomas develop mainly in the small intestine, whereas colorectal cancer is far more frequent in FAP patients. In the mouse, the second wild-type APC allele is systematically inactivated by homologous recombination with the mutated allele, leading to LOH [9], [36], [38], [39]. This lack of selective pressure for a secondary point mutation is contrasting to the human situation and indicates that the size and even the presence of APC are not important in the mouse models. It may reflect some functional difference between both APC orthologues. Alternatively, it might be that truncated APC becomes necessary at later stages of tumoural progression which the animals never reach because the adenomas limit their life span. In both cases, it follows that the contribution of truncated APC to human tumourigenesis is unlikely to be understood in the currently available mouse models, albeit they remain adequate tools to study the events consecutive to β-catenin stabilization, as recently demonstrated [14], [40].

As truncated APC seems required for the development of human colorectal cancer, it constitutes a suitable therapeutic target. Allele-specific RNA interference of APC allows in principle to envisage a patient-specific therapy for colorectal cancer, whereby only cells affected by a mutation in the APC gene will be hampered in their proliferation capacity upon acquisition of a shRNA matching the mutation [41], [42]. Allele-specific RNA interference using a shRNA specific of truncated APC expressed in the VACO4A cell line resulted in an inhibition of proliferation only in the VACO4A cell line containing the mutation matching the shRNA and not in all other cell lines. The success in producing a siRNA specific of a defined APC allele depended however on the nature of the APC mutation. There are several reports indicating that RNA interference can distinguish two alleles differing by a point mutation [43], but the allele distinction was better when wild-type and mutant APC alleles were differing by a deletion (data not shown), probably because the structure of the RNA duplex in the RNA-induced silencing complex [18] is more affected, as compared to the situation involving a point mutation. Thus, for a better chance of discrimination between wild-type and mutant APC, it seems preferable to choose two APC alleles differing by a deletion. Approximately one third of APC mutations are point mutations (36%), whereas the remaining two-thirds are either small insertions (12%) or, in most cases, deletions of one or several base pairs (51%) (visit www.umd.be:2070/ for a detailed description of APC mutations). The prevalence of insertions and deletions makes allele-specific RNA interference toward APC an attractive possibility, but the success in designing a siRNA specific of a defined APC mutation depends also on the sequence context where it is located. Indeed some deletions occur in repeated sequences of a low complexity that may not be adequate for allele-specific RNA interference. For this reason, it is difficult to predict how many different insertions and deletions would be suitable for targeting APC.

Together, we have shown that truncated APCs from colorectal cancer cells are required for cell proliferation in vitro and still keep a control over the transcriptional activity of β-catenin despite their structural variability. It remains to be determined how they exert these functions at the molecular level.

## Materials and Methods

### Cells

CaCo2, HT29, GP2D, LoVo, VACO4A and SW948 colorectal cancer cell lines [44] and HEK293T human embryonic kidney cells were maintained in DMEM medium (PAA Laboratories, Cölbe, Germany) supplemented with 10% foetal calf serum (Perbio Laboratories, Frankfurt/Main, Germany) and 1% penicillin and 1% streptomycin (PAA Laboratories) at 37°C in a humidified atmosphere with 10% CO_2_. For culturing VACO4A cells, Petri plates were coated with mouse collagen IV (50 µg/ml in PBS). APC mutations in the cell lines are described in [44] and the resulting truncated APC protein products are shown in fig. 1.

### Plasmids and shRNAs

The pSuper-based short hairpin RNA (shRNA) expression constructs were generated by cloning annealed oligonucleotides into the BglII/HindIII-digested pSUPER vector [45]. The N-APC and VACO4A specific shRNA sequences were 729 GAGGTCATCTCAGAACAAG and 4053 AGCTGTTGAA-----TTCAGGAGC, respectively. Numbers refer to the position of the first nucleotide in the APC open reading frame and the dashes show the VACO4A specific 5 base pairs deletion.

### Lentiviral vectors

Lentiviral plasmid pTRIPΔU3-EF1α/EGFP [46] was modified by inserting the linker 5′-aattcggttacctctagaactagtcccgggcatatggtcgacgctagcg-3′ at the unique EcoRI site (the modified vector is called pTRIPΔU3-EF1α/EGFP-LF). The APC shRNA expression cassettes were excised from the pSuper based APC shRNA expression plasmids (as XhoI-XbaI fragments) and sub-cloned between XbaI-SalI sites in pTRIPΔU3-EF1α/EGFP-LF.

### Lentiviral vector supernatants

Lentiviral vector particles were produced as described. [47] In brief, HEK293T cells were cotransfected with the lentiviral packaging (pCMV-dR8.2dvpr, 18 µg) and envelope (pMD.2G, 9 µg) plasmids, along with the lentiviral vector pTRIPΔU3-EF1α/EGFP-LF-shRNA (40 µg) encoding the shRNAs, in 15 cm culture dishes with polyethylenimine transfection reagent (1 mg/ml stock, 3 µl/µg DNA). Cells were washed 8 hours later. Viral supernatants were collected 48 to 60 hours after transfection, cleared by centrifugation (2000 g at room temperature), filtered through 0.45 µm filters and either applied directly onto the cell lines or frozen.

### Viral transduction protocol

The cell lines were plated and grown to 80% confluency. An infection cocktail consisting of the retroviral supernatant containing 8 µg/ml Sequabrene (Sigma-Aldrich, Steinheim, Germany) was added directly on the cells and infection was allowed to proceed for 24 hours, after which cells were washed and cultured further for 48 hours with fresh medium. GFP positive cells were sorted by fluorescence activated cell sorting (FACS) on a MoFlo apparatus (Beckman Coulter, Krefeld, Germany).

### Cell proliferation assay

A 3-(4,5-dimethylthiazol-2-yl)-2,5-diphenyl tetrazolium bromide (MTT) assay kit from Roche Diagnostics (Mannheim, Germany) was used to determine cell numbers according to the manufacturer's instructions.

### Cell extracts and western blotting

Cell extracts were prepared and western blotting was performed as described [1]. The antibodies used were APC antibody (ALi 12–28) from Abcam (Cambridge, UK), anti-β-actin from Santa Cruz Biotechnologies (Heidelberg, Germany), anti-α-tubulin from AbD Serotec (Düsseldorf, Germany), anti-Ecadherin from Sigma (Taufkirchen, Germany), anti-lamin from Santa Cruz Biotechnologies (Heidelberg, Germany) and secondary antibodies coupled to horseradish peroxidase from Dianova (Hamburg, Germany). The blots were developed using the chemiluminescence reagents Western Lightning™ from Perkin Elmer Life Sciences (Boston, USA) and the signals were detected under a LAS-3000-Fuji camera from Raytest (Straubenhardt, Germany).

### Nuclear extracts

Cells were washed two times with PBS and incubated for 10 min at room temperature in 10 mM HEPES, pH 7.9, 10 mM KCl, 0.1 mM EDTA, 0.4% IGEPAL CA630 (Sigma, Taufkirchen, Germany), 1 mM DTT and 1 mM PMSF. Lysates were harvested using a cell scraper and centrifuged at 4°C at top speed (15000×*g*) for 3 min. Supernatants and pellets were mixed with SDS-PAGE sample buffer to give the “cytoplasmic” and nuclear fractions, respectively.

### Semi-quantitative RT-PCR

Total cellular RNA was isolated using peqGold Trifast reagent from Peqlab (Erlangen, Germany), cDNAs were generated by reverse transcriptase reactions using AffinityScript™ QPCR cDNA Synthesis kit from Agilent Technologies Deutschland (Böblingen, Germany) and PCR was performed using Paq5000™ DNA Polymerase kit from Agilent Technologies Deutschland, according to manufacturer's instructions. The following thermal cycling parameters were used: 30 s at 95°C, 30 s at 55°C (for APC and Lgr5) or 57°C (for Axin2 and GAPDH), and 45 s at 72°C, with an initial step of 95°C for 3 min and a final step of 72°C for 5 min. Sequences of primers were as follows: APC, 5′-AAGTTGCGGCCGCTGGGAACCAAGGTGGAAATGGTG-3′ (forward) and 5′-AAGTCGCGGCCGCCTATTCCTATTCAACAGGAGCTGGCATTG-3′ (reverse); Axin2, 5′-GCAAACTTTCGCCAACCGTG-3′ (forward) and 5′ -CTCTGGAGCTGTTTCTTACTGCCC-3′ (reverse); Lgr5, 5′-CTTGGCCCTGAACAAAATACA-3′ (forward) and 5′- AAGGGTTGCCTACAAATGCTT- 3′ (reverse); GAPDH, 5′-CCTGCTTCACCACCTTCTTG -3′ (forward) and 5′-CTTCACCACCATGGAGAAGG-3′ (reverse).

### siRNA transfection

siAPC (aagacgttgcgagaagttgga) or siGFP (aagctacctgttccatggcca) (50–100 nM final concentration) were transfected into cells overnight using 1 µl Lipofectamine 2000 (Invitrogen, Darmstadt, Germany) per µl siRNA (20 µM). The sequences show only the coding strand.

### TOP/FOP reporter assays [48]

The TOPglow reporter consists of a tandem repeat of four TCF/LEF1 binding sites inserted in front of a TATA box [49], driving the expression of luciferase in a β-catenin-dependent manner. In the FOPglow reporter, the four binding sites are mutated to abolish binding of TCF/LEF1. pUHD16.1 plasmid expressing ß-galactosidase was transiently transfected together with either FOPglow or TOPglow plasmids at an equimolar ratio (300 ng each for SW948 using polyethylenimine transfection reagent (1 mg/ml stock, 3 µl/µg DNA) and 75 ng each for GP2D and CaCo2 using Lipofectamine 2000). The transcriptional activity measured 48 h post transfection is defined as the ratio of TOPglow or FOPglow luciferase values to the β-galactosidase values.

### Xenograft experiments

For xenograft experiments, 6×10^5^ HT29 cells infected with lentiviruses for expression of shVEC, shN-APC or shVACO4A were resuspended in 200 µl PBS and injected subcutaneously into the flanks of 6-week-old female BALB/c nu/nu mice (Charles River Laboratories, Wilmington, USA). Tumour size was measured once a week with a caliper and tumour volume was calculated using the formula: (length×width^2^)/2. Mice were sacrificed 6 weeks after inoculation or as soon as a reduction of vitality was observed. Experiments were performed according to the german animal protection law.

## Supporting Information

Figure S1 **HT29, LoVo and GP2D cells expressing either the sh-Vec or the sh-NAPC were fractionated into cytoplasmic and nuclear extracts.** Western blotting using anti-Ecadherin and anti-lamin antibodies reveals the quality of the preparations. β-catenin is shown above. The dotted lines indicate the removal of intervening lanes.
(PPT)

Figure S2 **Triton-X100 cell lysates from HCT116 cells expressing either the sh-VEC, the sh-N-APC or the shVACO4A were submitted to western blotting using either anti-APC or anti-β-actin antibodies.**
(PPT)

Figure S3 **Triton-X100 and hypotonic cell lysates derived from the same numbers of DLD1, HT29, LoVo and SW480 cells were submitted to western blotting using anti-APC and anti-β-catenin antibodies, respectively.**
(PPT)
