# Supplementary material for: A Common Role for Various Human Truncated Adenomatous Polyposis Coli Isoforms in the Control of Beta-Catenin Activity and Cell Proliferation
Source: PLoS One. 2012 Apr 3;7(4):e34479. doi: 10.1371/journal.pone.0034479 (PMC3317983; doi:10.1371/journal.pone.0034479)
Supplement: Figure S1 — HT29, LoVo and GP2D cells expressing either the sh-Vec or the sh-NAPC were fractionated into cytoplasmic and nuclear extracts. Western blotting using anti-Ecadherin and anti-lamin antibodies reveals the quality of the preparations. β-catenin is shown above. The dotted lines indicate the removal of intervening lanes. (PPT) [file pone.0034479.s001.ppt]

## Slide 1
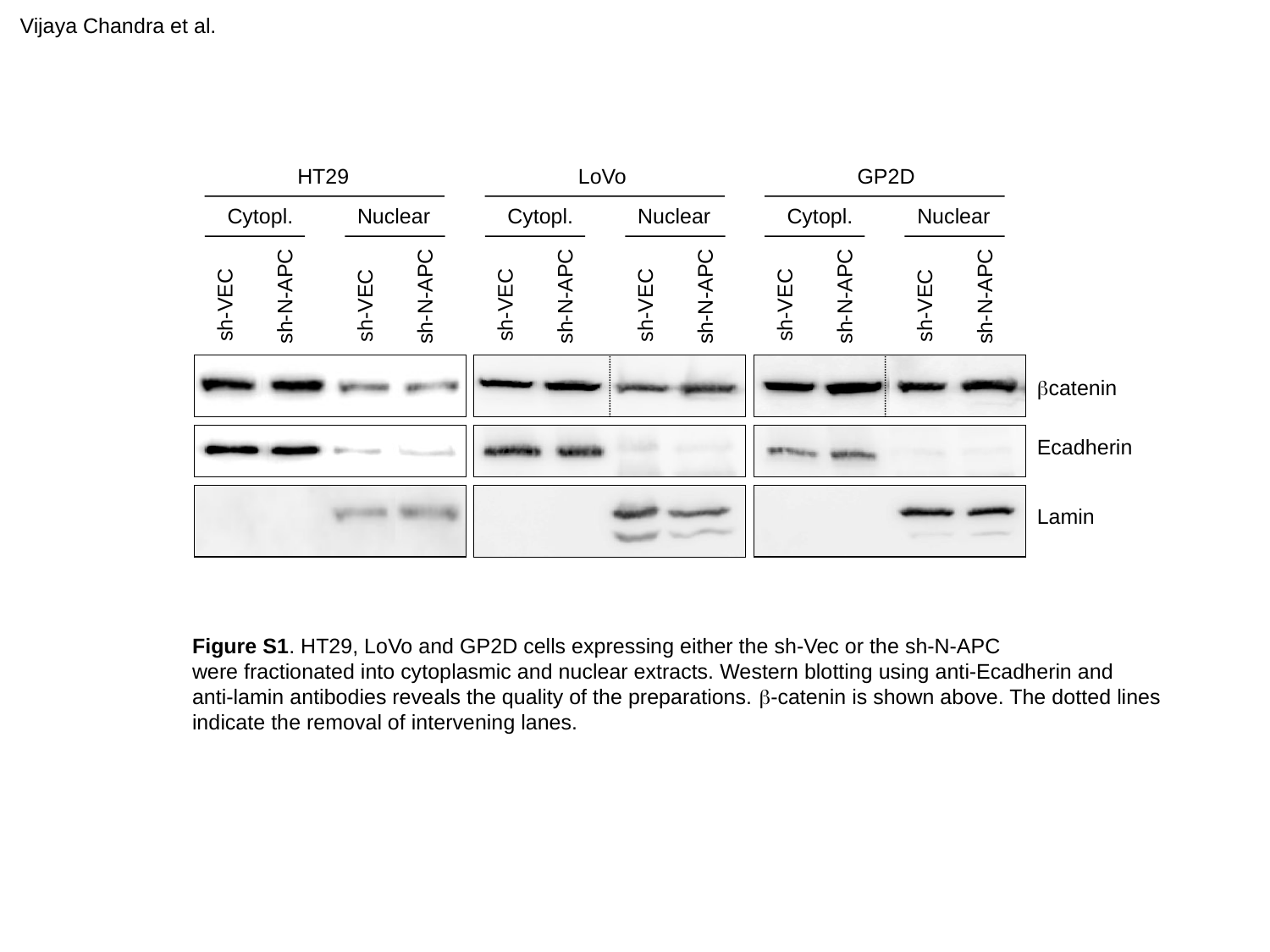

Vijaya Chandra et al.
HT29
LoVo
GP2D
Cytopl.
Nuclear
Cytopl.
Nuclear
Cytopl.
Nuclear
sh-N-APC
sh-N-APC
sh-N-APC
sh-N-APC
sh-N-APC
sh-N-APC
sh-VEC
sh-VEC
sh-VEC
sh-VEC
sh-VEC
sh-VEC
βcatenin
Ecadherin
Lamin
Figure S1. HT29, LoVo and GP2D cells expressing either the sh-Vec or the sh-N-APC
were fractionated into cytoplasmic and nuclear extracts. Western blotting using anti-Ecadherin and
anti-lamin antibodies reveals the quality of the preparations. β-catenin is shown above. The dotted lines
indicate the removal of intervening lanes.
