# Supplementary material for: A Common Role for Various Human Truncated Adenomatous Polyposis Coli Isoforms in the Control of Beta-Catenin Activity and Cell Proliferation
Source: PLoS One. 2012 Apr 3;7(4):e34479. doi: 10.1371/journal.pone.0034479 (PMC3317983; doi:10.1371/journal.pone.0034479)
Supplement: Figure S2 — Triton-X100 cell lysates from HCT116 cells expressing either the sh-VEC, the sh-N-APC or the shVACO4A were submitted to western blotting using either anti-APC or anti-β-actin antibodies. (PPT) [file pone.0034479.s002.ppt]

## Slide 1
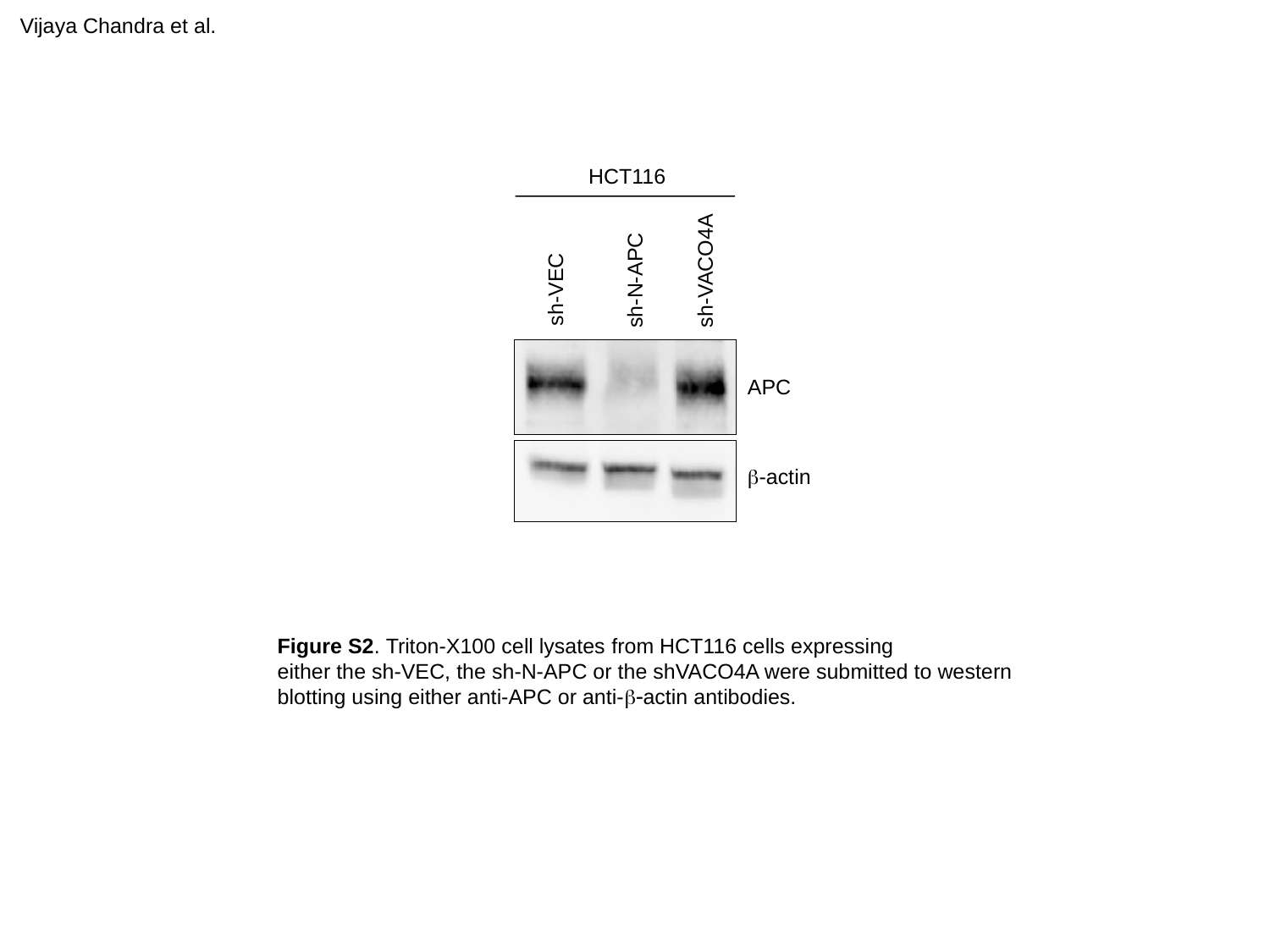

Vijaya Chandra et al.
HCT116
sh-VACO4A
sh-N-APC
sh-VEC
APC
β-actin
Figure S2. Triton-X100 cell lysates from HCT116 cells expressing
either the sh-VEC, the sh-N-APC or the shVACO4A were submitted to western
blotting using either anti-APC or anti-β−actin antibodies.
