# Supplementary material for: A Common Role for Various Human Truncated Adenomatous Polyposis Coli Isoforms in the Control of Beta-Catenin Activity and Cell Proliferation
Source: PLoS One. 2012 Apr 3;7(4):e34479. doi: 10.1371/journal.pone.0034479 (PMC3317983; doi:10.1371/journal.pone.0034479)
Supplement: Figure S3 — Triton-X100 and hypotonic cell lysates derived from the same numbers of DLD1, HT29, LoVo and SW480 cells were submitted to western blotting using anti-APC and anti-β-catenin antibodies, respectively. (PPT) [file pone.0034479.s003.ppt]

## Slide 1
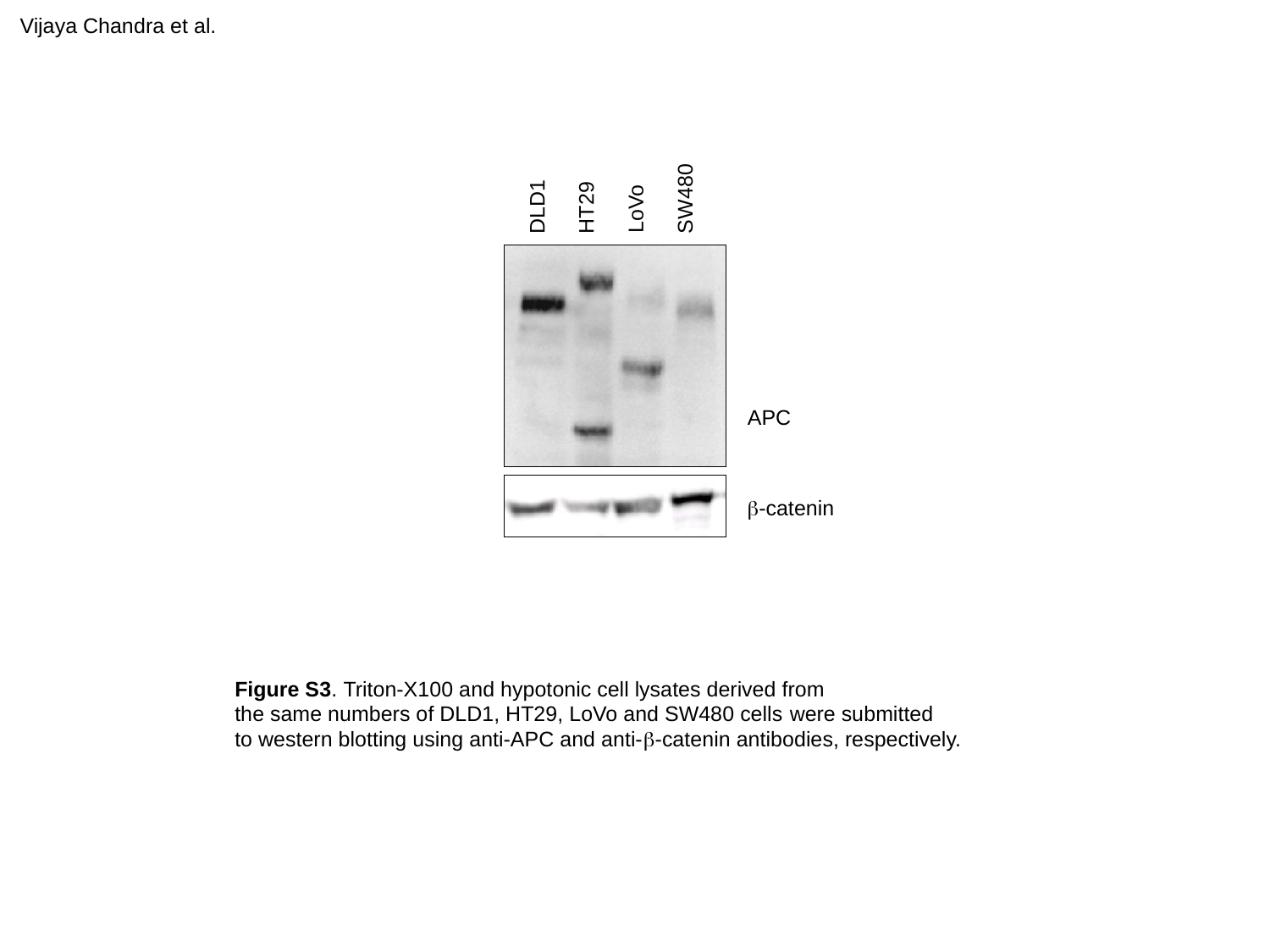

Vijaya Chandra et al.
SW480
DLD1
HT29
LoVo
APC
β-catenin
Figure S3. Triton-X100 and hypotonic cell lysates derived from
the same numbers of DLD1, HT29, LoVo and SW480 cells were submitted
to western blotting using anti-APC and anti-β-catenin antibodies, respectively.
